# A co-culture model with brain tumor-specific bioluminescence demonstrates astrocyte-induced drug resistance in glioblastoma

**DOI:** 10.1186/s12967-014-0278-y

**Published:** 2014-10-04

**Authors:** Ning Yang, Tao Yan, Huaiyang Zhu, Xiao Liang, Lina Leiss, Per Øystein Sakariassen, Kai Ove Skaftnesmo, Bin Huang, Daniela Elena Costea, Per Øyvind Enger, Xingang Li, Jian Wang

**Affiliations:** Department of Neurosurgery, Qilu Hospital of Shandong University, Jinan, China; Department of Biomedicine, University of Bergen, Bergen, Norway; Brain Science Research Institute, Shandong University, Jinan, China; The Gade Laboratory of Pathology, Department of Clinical Medicine, University of Bergen, Bergen, Norway; Department of Neurosurgery, Haukeland University Hospital, Bergen, Norway; Neuro Clinic, Haukeland University Hospital, Bergen, Norway

**Keywords:** Co-culture, Bioluminescent assay, Drug resistance, Glioblastoma

## Abstract

**Background:**

Although several studies suggest that stromal fibroblasts mediate treatment resistance in several cancer types, little is known about how tumor-associated astrocytes modulate the treatment response in brain tumors. Since traditionally used metabolic assays do not distinguish metabolic activity between stromal and tumor cells, and since 2-dimensional co-culture system does not recreate the formidable complexity of the microenvironment within 3-dimensional structures such as solid tumor tissue, we instead established a glioblastoma (GBM) cell-specific bioluminescent assay for direct measurements of tumor cell viability in the treatment of clinical relevant drugs.

**Methods:**

Using lentiviral transfection, we established a panel of human GBM cell lines constitutively expressing a fusion transgene encoding luciferase and the enhanced green fluorescence protein (eGFP). We then initiated co-cultures with immortalized astrocytes, TNC-1, and the eGFP/Luc GBM cell lines. Next, we treated all eGFP/Luc GBM cell lines with Temozolomide (TMZ) or Doxorubicin, comparing co-cultures of glioblastoma (GBM) cells and TNC-1 astrocytes with mono-cultures of eGFP/Luc GBM cells. Cell viability was quantitated by measuring the luciferase expression.

**Results:**

Titration experiments demonstrated that luciferase expression was proportional to the number of eGFP/Luc GBM cells, whereas it was not influenced by the number of TNC-1 cells present. Notably, the presence of TNC-1 astrocytes mediated significantly higher cell survival after TMZ treatment in the U251, C6, A172 cell lines as well as the *in vivo* propagated primary GBM tumor cell line (P3). Moreover, TNC-1 astrocytes mediated significantly higher survival after Doxorubicin treatment in the U251, and LN18 glioma cell lines.

**Conclusion:**

Glioma cell-specific bioluminescent assay is a reliable tool for assessment of cell viability in the brain tumor cell compartment following drug treatment. Moreover, we have applied this assay to demonstrate that astrocytes can modulate chemo sensitivity of GBM tumor cells. These effects varied both with the cell line and cytotoxic drug that were used, suggesting that several mechanisms may be involved.

## Background

Malignant tumors of the brain, in particular glioblastoma (GBM) represent a major unsolved clinical challenge [1]. Although the addition of radiotherapy and chemotherapy modestly prolong survival, gliomas are inherently resistant to these treatments, and the prognosis remains poor. All tumors eventually recur and median survival is about 14.6 months [2].

Although GBM stem cells have been implicated in brain tumorigenesis [3,4], studies suggest that other mechanisms are involved as well [5,6]. In other cancer types, studies clearly show that tumor-stroma interactions regulate multiple aspects of tumor biology, including tumor cell proliferation, invasion and drug resistance [7-9]. Stromal cells associated with malignant brain tumors include glial cells, endothelial cells, pericytes, immune cells and neurons, among which glial cells are the most abundant cell type in the brain [10]. As main subpopulation of glial cells, astrocytes have been shown to upregulate survival genes in brain metastatic tumor cells from melanoma and breast cancers, thereby mediating chemoresistance [11,12]. However, little is known whether astrocytes mediate similar effects in GBMs, and how astrocytes react in response to chemotherapy.

Previously, a tumor cell-specific platform has been introduced to study stroma-induced changes in sensitivity to anti-neoplastic drugs in various cancers using a 2-dimensional (2-D) co-culture system [13]. Although this is an extremely valuable tool that allows for high-throughput experiments, it does not recreate the formidable complexity of the microenvironment within 3-dimensiona (3-D) structures such as solid tumor tissue. Multicellular spheroids are 3-D structures that more accurately reflect the complex microenvironment in situ, compared to cells growing as monolayers. Notably, glioma spheroids initiated from human biopsies or glioma cell lines have been commonly used to study brain tumor biology in matrigel, collagen gels and in medium [14-16]. In this study, we established a 3-D model with floating co-culture spheroids of bioluminescent GBM tumor cells and non-luminescent rat astrocytic cells, as well as spheroid cultures of bioluminescent GBM tumor cells only. This tumor cell-specific bioluminescence allowed us to study how the presence of astrocytic cells modulated the response to clinically relevant drugs in a panel of GBM tumor cell lines.

## Methods

### Ethics statement

The collection of tumor tissue from patients was approved by the Regional Ethics Committee at Haukeland University Hospital (Project number 013.09; Bergen, Norway). All patients signed informed written consent. The protocol was approved by the Committee on the Ethics of Animal Experiments of the University of Bergen (Bergen, Norway).

### Cell culture and reagents

HF66 cells were established at Henry Ford Midwest Neuro-Oncology Center, Detroit, MI [17]; A172, U251, LN18, C6, TNC-1 were purchased from the American Tissue Culture Collection (ATCC; Manassas, VA, USA). All the cells were cultured in Dulbeccos’ modified Eagles medium (Sigma-Aldrich, St. Louis, MO, USA) containing 10% fetal bovine serum, supplemented with non-essential amino acids, 100 U/ml Penicillin/Streptomycin, 400 μM L-glutamine, all from Cambrex (Cambrex, East Rutherford, NJ, USA). P3 GBM cells were taken and cultured from a patient biopsy. All cell lines were maintained at 37°C in a 5% CO2-humidified atmosphere. Temozolomide (TMZ) was purchased from Tocris Bioscience and dissolved in Dimethyl Sulfoxide. Doxorubicin was purchased from Pharmacia & Upjohn and dissolved in sterile water.

### Lentiviral vector production and infection of cells by lentiviral vectors

The packaging plasmid psPAX.2 and enveloping plasmid pMD2.G were kindly provided by Dr. Didier Trono's laboratory (CMU, Geneva, Switzerland). The lentiviral eGFP/Luciferase (Luc) vector and the viral infection protocol were kindly provided by Irving Weissman’s laboratory (Stanford University, USA) [18]. Lentivirus was produced by BBS/CaCl2 mediated triple-transfection of 293FT cells with psPAX.2, pMD2.G, and eGFP/Luc vectors. Lentiviral particles were harvested 48 h and 72 h post-transfection, and were subsequently sterile filtered. Viral infection was performed by centrifugation of cells at 2225 rpm for 90 minutes at room temperature in the presence of 10 μg/mL polybrene (Sigma-Aldrich). The infected tumor cells were kept in culture and expanded for late purification by fluorescence-activated cell sorting.

### Fluorescence-activated cell sorting

Lentivirally infected A172, U251, LN18, HF66, C6 and P3 tumor cells harboring eGFP and luciferase were trypsinized and washed with ice-cold FACS buffer (PBS with 2% FBS). The cell suspension was then centrifuged at a speed of 300 G for 5 min (4°C). The cell pellets were re-suspended in FACS buffer and then filtered through a 40-μm cell strainer in order to remove any clumping cells before sorting. The cells were sorted using a cell sorter (FACS Aria SORP, BD Biosciences, Erembodegem, Belgium) on the basis of single cell viability and eGFP expression. The separation was confirmed by fluorescence microscopy (Nikon ellipse 2000, Nikon, Japan).

### Spheroids formation

Trypsinized eGFP/Luc GBM cells were seeded into each well of a 96 well plate, either non-transparent (Tomtec plastics KFT, Hungary) or transparent V-shape well plates (NUNC, Thermo, MA, USA) in the presence or absence of pre-plated TNC-1 cell, with 100 μl culture medium containing concentration of 1.74 μg/mL methylcellulose (Sigma-Aldrich). Subsequently, the 96-well plates were spun down at 756 G at room temperature for 15 minutes in order to let cells form an aggregate in the well. The plates were then cultured in a standard tissue culture incubator with 5% CO2 in air and 100% relative humidity at 37°C. Spheroids derived from the mixture of 8000 eGFP/Luc GBM cells and 5000 TNC-1 cells and 8000 eGFP/Luc GBM cells only were chosen in the MTS assay and Bioluminescence assay. Spheroids were photographed at × 4 magnification using the BD Pathway Bioimager (BD Pathway 855, BD Biosciences, CA, USA).

### MTS assay

(3-(4,5-dimethylthiazol-2-yl)-5-(3-carboxymethoxyphenyl)-2-(4-sulfophenyl)-2H-tetrazolium) (MTS) assay was carried out according to a previously described method with slight modifications [19]. Briefly, after culturing the spheroids in the incubator for 2 days, different concentrations of TMZ were added to the spheroids, depending on the IC50 dose for the cell lines. After incubating with TMZ for 5 days, the MTS substrate (Promega, CA, USA) was added to each well. Following 3 hours of incubation, the absorbance was analyzed at 490 nm on a spectrophotometer (Asys UVM 340, Biochrom, MA, USA). The experiments were performed 3 times.

### Spheroids dissociation and flow cytometry

The spheroids that were treated by a serial diluted concentration of TMZ in MTS and bioluminescence assay groups were collected respectively and washed in 1× phosphate buffered saline following a serial trypsinization to obtain single cell suspension. Cells were then analyzed by Accuri C6 (BD Biosciences, Erembodegem, Belgium), in which eGFP/Luc positive tumor cells were counted.

### Bioluminescence assay

Bioluminescence assay experiments were conducted both on spheroids treated by TMZ and on spheroids treated by Doxorubicin, modified after a previously described method [13]. Briefly, after culturing pre-made spheroids in the 96-wells plate for 2 days, different concentrations of TMZ/Doxorubicin were added into each well with a total volume of 100 μl medium. After 5 days incubation of the spheroids with TMZ/Doxorubicin, the luciferin substrate was added to the wells and luciferase activity was then measured at different time intervals using a PerkinElmer Victor 3 Multilabel plate reader (PerkinElmer, CA, USA). Luciferin substrate solution was prepared by dissolving firefly diluent (Biosynth, Switzerland) in ddH2O supplemented with 100 nM/L Adenosine 5’Triphosphate (ATP, Sigma-Aldrich) to a stock concentration of 3×10^4^ μg/ml and their final concentration in each well was 375 μg/mL. All experiments were performed 3 times.

### Statistics

We assessed the correlation between cell number and bioluminescence by estimating the coefficient of determination (R^2^). Growth curves were analyzed using the two-way ANOVA measures, and *p*-values < 0.05 were considered significant.

## Results

### Bioluminescence intensity of spheroids is reproducible and proportional with the number of tumor cells

**Figure 1:**
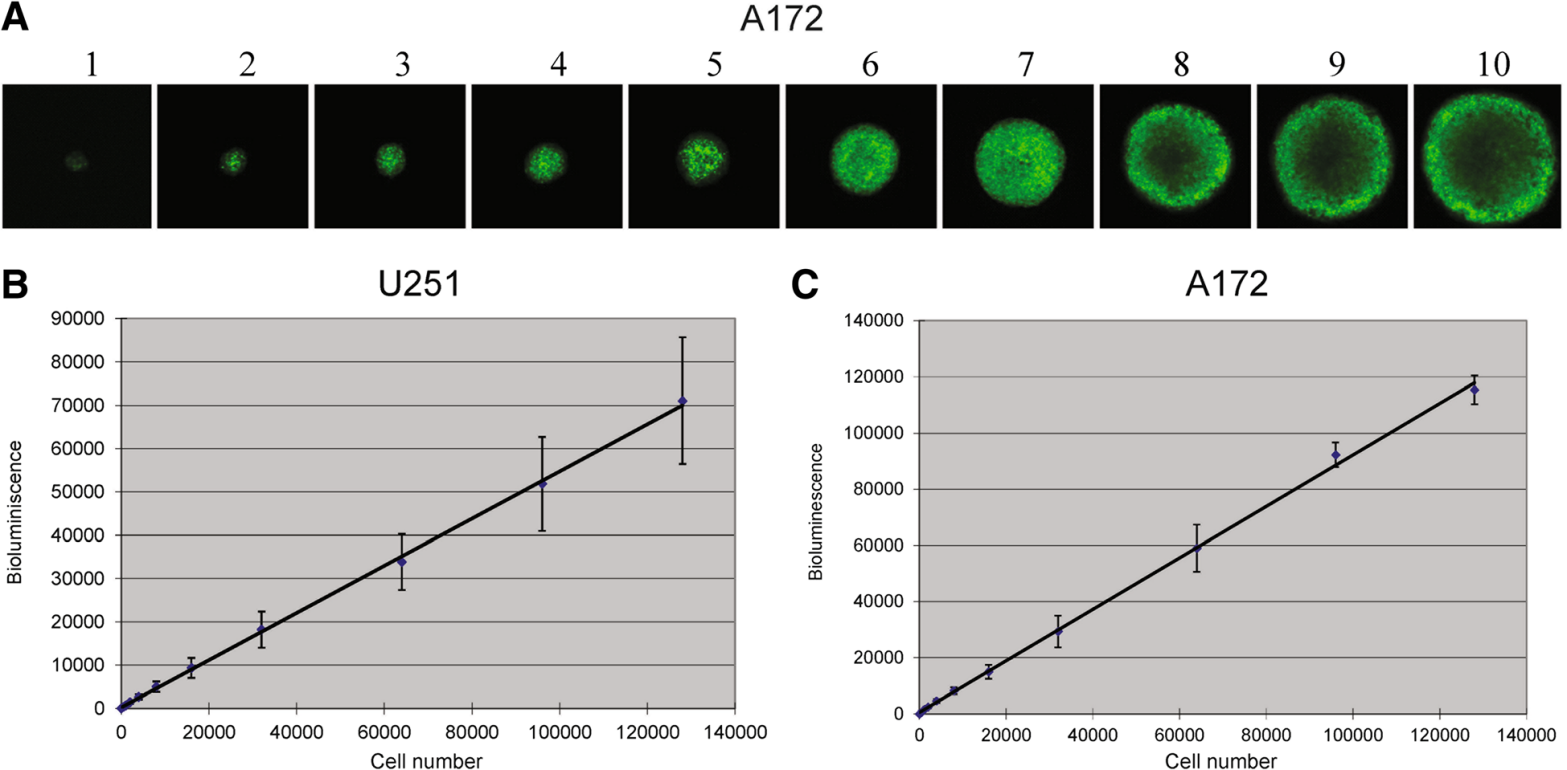
**Bioluminescence expression is proportional to the number of tumor cells in spheroids formation. A**: eGFP expressing glioma spheroids initiated from an increasing number of 172 glioma cells (500, 1000, 2000, 4000, 8000, 16000, 32000, 64000, 96000, 128000) per well. **B** and **C**: Bioluminescence measurements for increasing cell numbers of U251 and A172 glioma cells demonstrated a linear relationship (R^2^ = 0.9993 for U251 cells and R^2^ = 0.9987 for A172 cells).

Spheroids of GBM tumor cells only were initiated by seeding increasing numbers of tumor cells into individual wells of a 96-well plate (Figure 1A). Notably, comparison of different tumor cell numbers and bioluminescence values of the spheroids demonstrated a linear relationship (R^2^ = 0.9993 for U251 cells and R^2^ = 0.9987 for A172 cells) (Figure 1B and C). Thus, bioluminescence intensities were proportional to the number of tumor cells, demonstrating that bioluminescent could be used as a highly reliable measurement to quantitate the cell number.

### Tumor cell specific bioluminescence intensity is independent of the size of the TNC-1 stromal cell compartment

**Figure 2:**
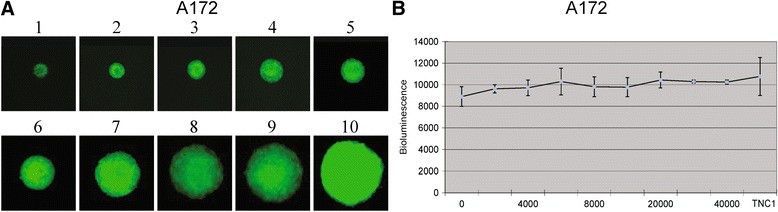
**TNC-1 cells have no influence on bioluminescence expression of tumor cells. A**: mixing a varying number of A172 eGFP/Luc tumor cells (500, 800, 1600, 3200, 6400, 8000, 16000, 32000, 48000 and 64000) with a varying number of stromal TNC-1 cells (100, 500, 1000, 2000, 4000, 56000, 810000, 210000, 200000, 30000 and 40000) respectively, initiate homogeneous spheroids. **B**: 8000 A172 eGFP/Luc tumor cells were co-cultured with TNC-1 with an increasing cell numbers (0, 500, 1000, 2000, 4000, 6000, 8000, 10000, 200000, 30000 and 40000) demonstrated TNC-1 has no influence on bioluminescence value changes of A172 tumor cells indicating bioluminescence intensity is proportional to tumor cells in the co-culture spheroids.

In order to investigate whether the number of stromal cells impacted on the bioluminescent signal detected from the tumor cell compartment, we established co-culture spheroids. By mixing a varying number of eGFP/Luc A172 tumor cells with a varying number of stromal TNC-1 cells, we obtained spheroids with a spatial distribution of the eGFP signal with different ratios of glial stromal cells and glioma cells (Figure 2A). The most homogeneous spheroids resulted from mixing 8000 tumor cells with 5000 TNC-1 cells, and these were used in subsequent treatent experiments. Moreover, bioluminescence intensities were measured from spheroids, mixing 8000 eGFP/Luc A172 tumor cells with a varying number of TNC-1 cells (0, 2000, 4000, 6000, 8000, 10000, 20000, 30000, 40000 and 50000), and demonstrated that the number of TNC-1 cells did not influence the quantification of tumor cell numbers (Figure 2B).

### Measurement of tumor-specific bioluminescence demonstrates reduction in cell survival rates not detected by the MTS assay (*p* < 0.05)

**Figure 3:**
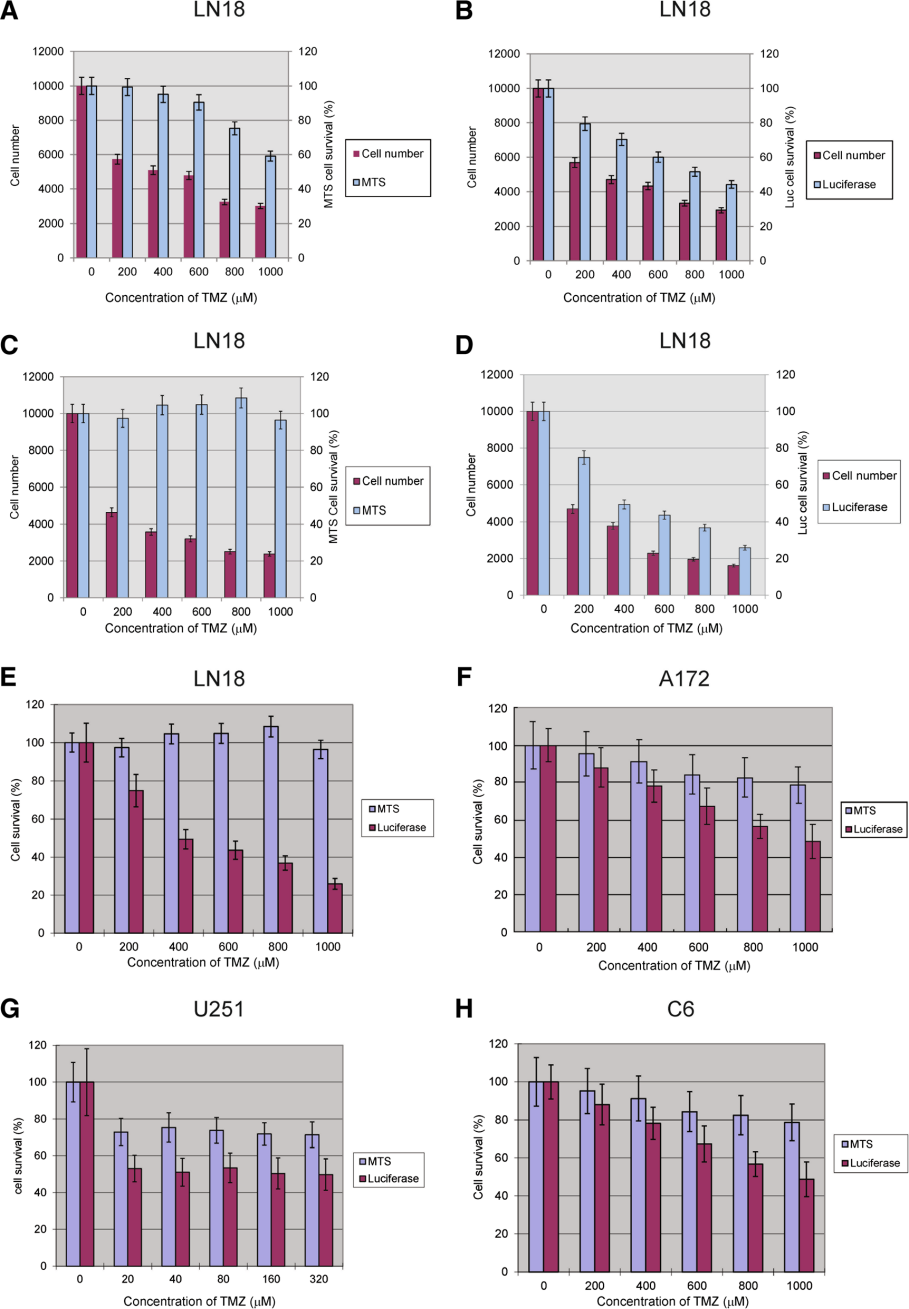
**Cell viability measured by bioluminescence versus the MTS assay after treatment with TMZ. A** and **B**: LN18 eGFP positive cells on mono-culture spheroids treated with TMZ at doses as indicated were counted by flow cytometry, and compared to measurements by MTS assay and bioluminescence assay, respectively. **C** and **D**: LN18 eGFP positive cells on co-culture spheroids with TNC-1 cells treated with TMZ at doses as indicated were counted by flow cytometry, and compared to measurements by MTS assay and bioluminescence assay, respectively. **E**, **F**, **G** and **H**: Comparison of MTS with bioluminescence intensity measurements in the LN18, A172, U251 and C6 glioma cell lines co-cultured with TNC-1 cells after treatment with TMZ at doses as indicated.

Next, cell numbers in GFP/Luc LN18 glioma cells treated with different doses of TMZ for five days were counted using flow cytometry. As expected, we observed a dose-dependent reduction in cell numbers reflecting cytotoxicity. We then compared measurements by the luciferase assay with the commonly used MTS assay on mono-culture and co-culture spheroids following treatment with different concentrations of TMZ. Results from these experiments demonstrated that tumor cell survival measured by both MTS assay and bioluminescence assay on mono-culture spheroids treated with different doses of TMZ showed dose-dependent cell survival rates as the reduction in cell numbers as determined by flow cytometry (Figure 3A and B). However, on co-culture spheroids treated with different doses of TMZ, MTS assay showed no dose-dependent cell survival rates as reflected the reduction in cell numbers as determined by flow cytometry, whereas bioluminescence assay showed significant dose-dependent cell survival rates mirrored the reduction in cell numbers as determined by flow cytometry (Figure 3C and D). Moreover, except for LN18, in the A172, U251 and C6 eGFP/Luc glioma cell lines we observed a consistent dose-dependent reduction in bioluminescence assay after TMZ treatment, whereas MTS showed no significant changes for any of these cell lines (Figure 3E, F, G and H).

### TNC-1 cells modulate sensitivity to TMZ in glioma cell lines

**Figure 4:**
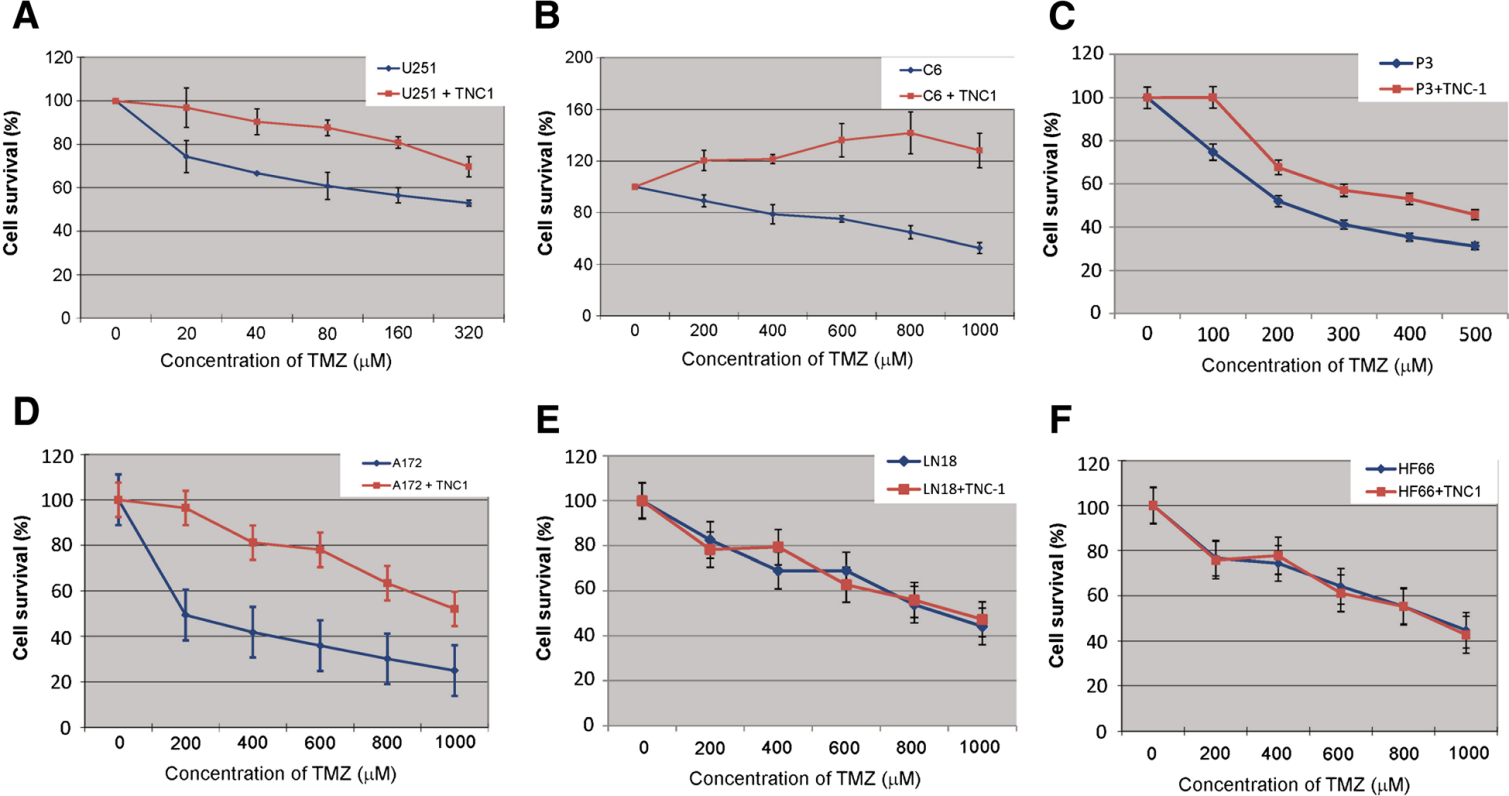
**TNC-1 cells modulate sensitivity of TMZ to GBM tumor cells. A**, **B**, **C** and **D**: TNC-1 attenuated the response of U251, C6, P3 and A172 tumor cells to TMZ treatment respectively. **E** and **F**: The cell survival of LN18 and HF66 to TMZ was not significantly different in the presence or absence of TNC-1.

We then tested the response of tumor cells to TMZ in the presence or absence of TNC-1 cells. Bioluminescence based quantification demonstrated that TNC-1 cells attenuated the response of the U251, C6, P3 and A172 eGFP/Luc glioma cell lines to TMZ at the concentration of 20–320 μM for U251; 200–1000 μM for C6 and A172; 100–500 μM for P3 (*p* < 0.05, Figure 4A, B, C and D). For the LN18 and HF66 glioma cell lines however, the TNC-1 cell line did not impact on chemosensitivity (Figure 4E and F) at the concentration of 200–1000 μM.

### TNC-1 cells modulate sensitivity to Doxorubicin in glioma cell lines

**Figure 5:**
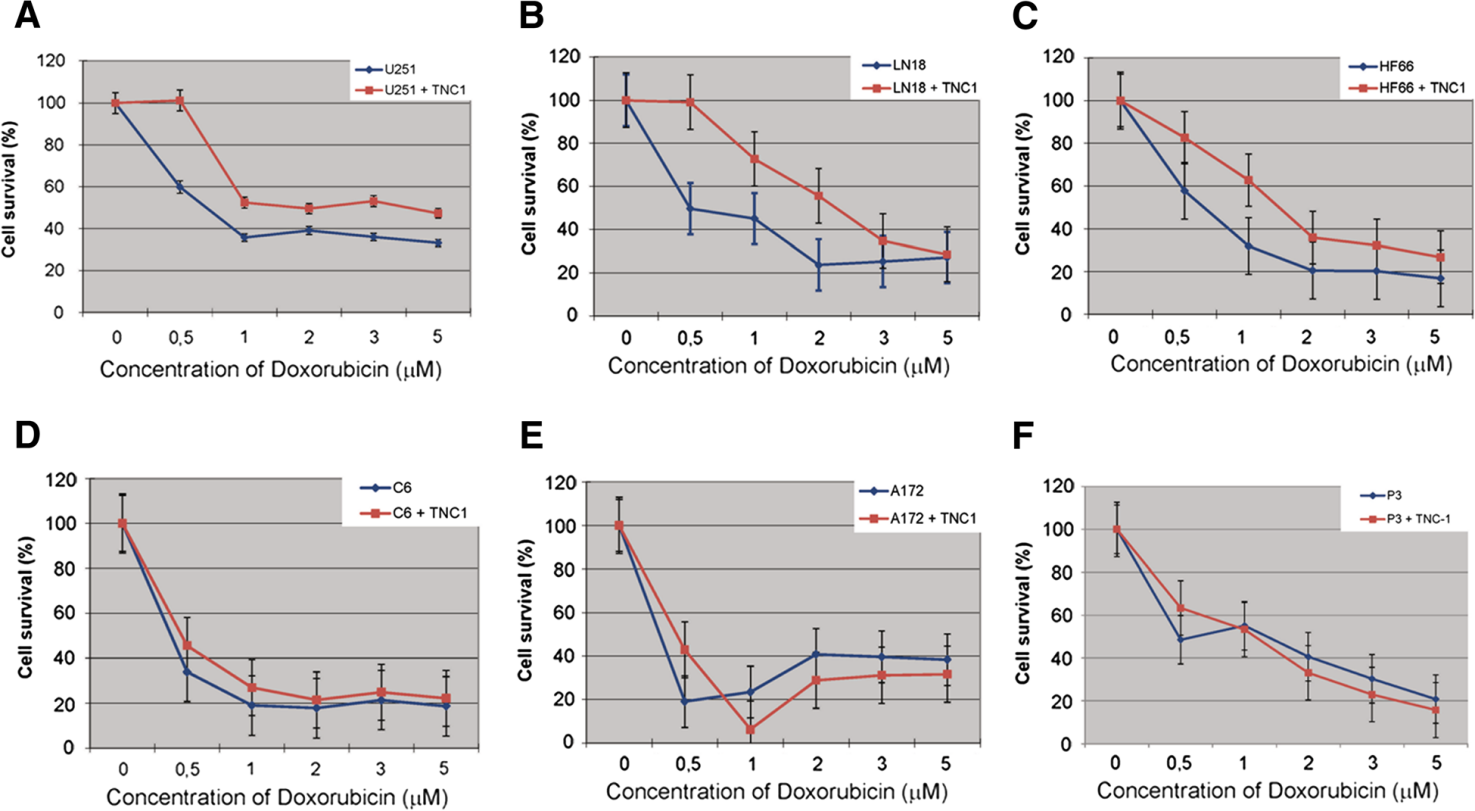
**TNC-1 cells modulate the sensitivity of Doxorubicin to GBM tumor cells. A**, **B** and **C**: TNC-1 attenuated the response of U251 and LN18 to Doxorubicin at the concentration of 0.5, 1 and 2 μM. HF66 seemed to be protected by TNC-1 at different concentration of doxorubicin, but not significant (*p* > 0.05) except at the concentration of 1 μM (*p* < 0.05). **D**, **E** and **F**: TNC1 has no effect on C6, A172 and P3 tumor cells. However, with increasing concentrations of TMZ more A172 cells were eliminated in the presence of TNC-1 compared to spheroids of tumor cells only.

Since Doxorubicin has shown efficacy in some studies against glioma using local delivery [20], we also tested whether the sensitivity of GBM cells to Doxorubicin was modulated by the presence of TNC-1 astrocytic cells. Again, the presence of TNC-1 attenuated the response of U251 and LN18 tumor cells to Doxorubicin as measured by bioluminescence intensity (Figure 5A and B), whereas no effects were seen for the C6, P3 and A172 glioma cell lines (Figure 5D, E and F). The effect of Doxorubicin at the concentration of 0.5, 1 and 2 μM were significantly attenuated by TNC-1 (*p* < 0.05) in the U251 and LN18 glioma cell lines. HF66 showed higher cell survival in the presence of TNC-1 cells although this was not significant except at a dose of 1 μM Doxorubicin (*p* < 0.05) (Figure 5C). Furthermore, with increasing concentrations of Doxorubicin more A172 cells were eliminated in the presence of TNC-1 compared to spheroids of tumor cells only.

## Discussion

Currently, little is known about the role of astrocytes in brain tumor in response to chemotherapy. Few models for drug testing take into account the role of tumor-astrocyte interactions in modulating treatment response in brain tumors for instance, how the presence of astrocytic cells modulated the chemo-response in GBMs, the most malignant brain tumors in human with short survivals. On the contrary, traditional chemotherapeutics testing *in vitro* is usually performed in pure tumor cell cultures. Moreover, these model systems usually involve monolayers and suspension tumor cell cultures. In this context, cells are growing on the surface of artificial plastic or glass substrate and in contact with other cells only at their periphery. Apparently, these models do not reflect the multicellular microenvironment found in the body. Although, expensive animal models have been accepted to use in drug testing by researchers, the best preclinical model should be relatively inexpensive, amenable to high-throughput screening, and most importantly, reflect human tumor biology as closely as possible. Previous studies have shown that 3-D model systems such as spheroid models reflect the biology of GBM closer than 2-D model systems such as monolayer cell cultures [21,22]. In order to address these limitations, in the current studies, we established a 3-D co-culture model comprising both TNC-1 astrocytic cells and different eGFP-luciferase expressing glioma cell lines. This model provides a standardized method for obtaining multiple cellular spheroids of equal size with a reproducible number of cells in a high-throughput manner.

MTS assay is well established and widely accepted for assessing cell viability [23]. In this study however, comparison of the MTS and bioluminescence assay, demonstrated that although both MTS assay and bioluminescence assay are reliable to measure the cell survival on mono-culture spheroids, measurement of bioluminescence intensity reflected alterations in cell survival more accurately on co-culture spheroid systems. A likely explanation for this finding is that the TNC-1 stromal cells contribute to the overall metabolic activity which is measured in the MTS assay, whereas the luciferase expression is restricted to the tumor cell compartment.

3-D spheroid systems provide a rapid, reproducible and high-throughput screen assay for the effective triaging of drug candidates prior to *in vivo* studies [24]. Furthermore, TMZ is now established as a primary treatment for GBM together with radiotherapy and surgery [25,26]. Doxorubicin has been reported to significantly improve survival when used for treatment of brain tumor in a 9 L glioma animal model by local delivery, and the chemo sensitivity in the animal after systemic injection was dramatically enhanced if Doxorubicin was coated with nanoparticles [20]. Despite this, the effects of Doxorubicin on patients have been generally disappointing. Thus we used the 3-D model to investigate how astrocytes impact on glioma cell sensitivity to TMZ and Doxorubicin. Strikingly, we found that the presence of TNC-1 astrocytic cells attenuated the anti-glioma efficacy of TMZ as well as Doxorubicin in several glioma cell lines. GBM tumor cells behaved differently in response to TMZ and Doxorubicin in the co-culture than when the tumor cells were cultured alone. In recent years, cancer associated fibroblast (CAF) mediated drug resistance has been extensively studied. Previously, McMillin et al. reported, using their co-culture 2-D model that tumor cells responded heterogeneously depending on the tumor type, accessory cells and cytotoxic reagents that were used [13]. Moreover, their molecular profiling studies in a multiple myeloma cell line interacting with stroma, suggested activation of pathways related to drug resistance. CAFs have been shown to protect breast cancer cells against apoptosis induced by Doxorubicin and the PARP-1 inhibitor ABT-888 [27]. Using a combined experimental model and theoretical approach, Falch et al. showed CAFs contributed to tumor growth and treatment resistance in melanoma [28]. In the normal state, as the most abundant subpopulation of glial cells, the roles of astrocytes in the brain overlap with the functions of fibroblasts in other organs, such as providing structural support. Thus, it is conceivable that astrocytes and fibroblasts also have similar roles in tumor progression. Ultimately, in light of our findings that TNC-1 mediated TMZ and Doxorubicin resistance in various GBM cell lines, we believe that the interaction of astrocytes and tumor cells can fundamentally affect both brain tumor growth and treatment and should be an important focus for future brain tumor research.

## Conclusions

We constructed a 3-D model, containing both the tumor and astrocyte cell compartments. In this model we co-cultured luciferase expressing tumor cells with luciferase-negative TNC-1 cells, so that the viability of tumor cells could be distinguished from accessory cells. Using this model, we demonstrate that TNC-1 cells may impact on sensitivity to TMZ and Doxorubicin in a cell line specific manner. We believe that co-culture 3-D spheroids could act as a preclinical high-throughput drug-screening model which may potentially reduce the cost for extensive testing *in vivo*. Moreover, this model may provide a valuable tool for investigating mechanisms underlying astrocytes-induced drug resistance.

## References

[1] CBTURS: **CBTRUS Statistical Report: Primary Brain and Central Nervous System Tumors Diagnosed in the United States in 2004–2006.** 2010. 10.1093/neuonc/not151 PMC3798196 24137015

[2] Stupp R, Mason WP, van den Bent MJ, Weller M, Fisher B, Taphoorn MJ, Belanger K, Brandes AA, Marosi C, Bogdahn U, Curschmann J, Janzer RC, Ludwin SK, Gorlia T, Allgeier A, Lacombe D, Cairncross JG, Eisenhauer E, Mirimanoff RO, European Organisation for Research and Treatment of Cancer Brain Tumor and Radiotherapy Groups, National Cancer Institute of Canada Clinical Trials Group (2005). Radiotherapy plus concomitant and adjuvant temozolomide for glioblastoma. N Engl J Med.

[3] Bao S, Wu Q, McLendon RE, Hao Y, Shi Q, Hjelmeland AB, Dewhirst MW, Bigner DD, Rich JN (2006). Glioma stem cells promote radioresistance by preferential activation of the DNA damage response. Nature.

[4] Singh SK, Hawkins C, Clarke ID, Squire JA, Bayani J, Hide T, Henkelman RM, Cusimano MD, Dirks PB (2004). Identification of human brain tumour initiating cells. Nature.

[5] Prestegarden L, Enger PO (2010). Cancer stem cells in the central nervous system–a critical review. Cancer Res.

[6] Prestegarden L, Svendsen A, Wang J, Sleire L, Skaftnesmo KO, Bjerkvig R, Yan T, Askland L, Persson A, Sakariassen PO, Enger PO (2010). Glioma cell populations grouped by different cell type markers drive brain tumor growth. Cancer Res.

[7] Fidler IJ (2003). The pathogenesis of cancer metastasis: the ‘seed and soil’ hypothesis revisited. Nat Rev Cancer.

[8] Mueller MM, Fusenig NE (2004). Friends or foes - bipolar effects of the tumour stroma in cancer. Nat Rev Cancer.

[9] Paget S (1989). The distribution of secondary growths in cancer of the breast 1889. Cancer Metastasis Rev.

[10] Charles NA, Holland EC, Gilbertson R, Glass R, Kettenmann H (2011). The brain tumor microenvironment. Glia.

[11] Lin Q, Balasubramanian K, Fan D, Kim SJ, Guo L, Wang H, Bar-Eli M, Aldape KD, Fidler IJ (2010). Reactive astrocytes protect melanoma cells from chemotherapy by sequestering intracellular calcium through gap junction communication channels. Neoplasia.

[12] Kim SJ, Kim JS, Park ES, Lee JS, Lin Q, Langley RR, Maya M, He J, Kim SW, Weihua Z, Balasubramanian K, Fan D, Mills GB, Hung MC, Fidler IJ (2011). Astrocytes upregulate survival genes in tumor cells and induce protection from chemotherapy. Neoplasia.

[13] McMillin DW, Delmore J, Weisberg E, Negri JM, Geer DC, Klippel S, Mitsiades N, Schlossman RL, Munshi NC, Kung AL, Griffin JD, Richardson PG, Anderson KC, Mitsiades CS (2010). Tumor cell-specific bioluminescence platform to identify stroma-induced changes to anticancer drug activity. Nat Med.

[14] Del Duca D, Werbowetski T, Del Maestro RF (2004). Spheroid preparation from hanging drops: characterization of a model of brain tumor invasion. J Neurooncol.

[15] Vinci M, Gowan S, Boxall F, Patterson L, Zimmermann M, Court W, Lomas C, Mendiola M, Hardisson D, Eccles SA (2012). Advances in establishment and analysis of three-dimensional tumor spheroid-based functional assays for target validation and drug evaluation. BMC Biol.

[16] Smith SJ, Wilson M, Ward JH, Rahman CV, Peet AC, Macarthur DC, Rose FR, Grundy RG, Rahman R (2012). Recapitulation of tumor heterogeneity and molecular signatures in a 3D brain cancer model with decreased sensitivity to histone deacetylase inhibition. PLoS One.

[17] Knott JC, Mahesparan R, Garcia-Cabrera I, Bolge Tysnes B, Edvardsen K, Ness GO, Mork S, Lund-Johansen M, Bjerkvig R (1998). Stimulation of extracellular matrix components in the normal brain by invading glioma cells. Int J Cancer.

[18] Willingham SB, Volkmer JP, Gentles AJ, Sahoo D, Dalerba P, Mitra SS, Wang J, Contreras-Trujillo H, Martin R, Cohen JD, Lovelace P, Scheeren FA, Chao MP, Weiskopf K, Tang C, Volkmer AK, Naik TJ, Storm TA, Mosley AR, Edris B, Schmid SM, Sun CK, Chua MS, Murillo O, Rajendran P, Cha AC, Chin RK, Kim D, Adorno M, Raveh T (2012). The CD47-signal regulatory protein alpha (SIRPa) interaction is a therapeutic target for human solid tumors. Proc Natl Acad Sci U S A.

[19] Malich G, Markovic B, Winder C (1997). The sensitivity and specificity of the MTS tetrazolium assay for detecting the in vitro cytotoxicity of 20 chemicals using human cell lines. Toxicology.

[20] Lesniak MS, Upadhyay U, Goodwin R, Tyler B, Brem H (2005). Local delivery of doxorubicin for the treatment of malignant brain tumors in rats. Anticancer Res.

[21] Mehta G, Hsiao AY, Ingram M, Luker GD, Takayama S (2012). Opportunities and challenges for use of tumor spheroids as models to test drug delivery and efficacy. J Control Release.

[22] Griffith LG, Swartz MA (2006). Capturing complex 3D tissue physiology in vitro. Nat Rev Mol Cell Biol.

[23] Cory AH, Owen TC, Barltrop JA, Cory JG (1991). Use of an aqueous soluble tetrazolium/formazan assay for cell growth assays in culture. Cancer Commun.

[24] Friedrich J, Seidel C, Ebner R, Kunz-Schughart LA (2009). Spheroid-based drug screen: considerations and practical approach. Nat Protoc.

[25] Nagasawa DT, Chow F, Yew A, Kim W, Cremer N, Yang I (2012). Temozolomide and other potential agents for the treatment of glioblastoma multiforme. Neurosurg Clin N Am.

[26] Tatar Z, Thivat E, Planchat E, Gimbergues P, Gadea E, Abrial C, Durando X (2013). Temozolomide and unusual indications: review of literature. Cancer Treat Rev.

[27] Martinez-Outschoorn UE, Goldberg A, Lin Z, Ko YH, Flomenberg N, Wang C, Pavlides S, Pestell RG, Howell A, Sotgia F, Lisanti MP (2011). Anti-estrogen resistance in breast cancer is induced by the tumor microenvironment and can be overcome by inhibiting mitochondrial function in epithelial cancer cells. Cancer Biol Ther.

[28] Flach EH, Rebecca VW, Herlyn M, Smalley KS, Anderson AR (2011). Fibroblasts contribute to melanoma tumor growth and drug resistance. Mol Pharm.

